# Numerically Exact Solution for a Real Polaritonic System under Vibrational Strong Coupling in Thermodynamic Equilibrium: Loss of Light–Matter Entanglement and Enhanced Fluctuations

**DOI:** 10.1021/acs.jctc.3c00092

**Published:** 2023-11-16

**Authors:** Dominik Sidler, Michael Ruggenthaler, Angel Rubio

**Affiliations:** † Max Planck Institute for the Structure and Dynamics of Matter and Center for Free-Electron Laser Science, Luruper Chaussee 149, Hamburg 22761, Germany; ‡ The Hamburg Center for Ultrafast Imaging, Luruper Chaussee 149, Hamburg 22761, Germany; § Center for Computational Quantum Physics, Flatiron Institute, 162 Fifth Avenue, New York, New York 10010, United States; ∥ Nano-Bio Spectroscopy Group, University of the Basque Country (UPV/EHU), San Sebastián 20018, Spain

## Abstract

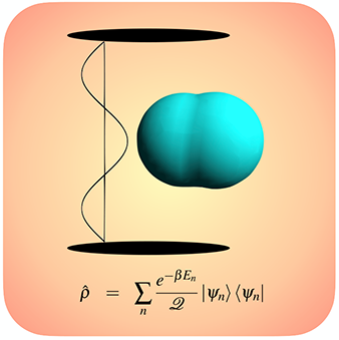

The first numerically exact simulation of a full ab initio molecular quantum system (HD^+^) under strong ro-vibrational coupling to a quantized optical cavity mode in thermal equilibrium is presented. Theoretical challenges in describing strongly coupled systems of mixed quantum statistics (bosons and Fermions) are discussed and circumvented by the specific choice of our molecular system. Our numerically exact simulations highlight the absence of zero temperature for the strongly coupled matter and light subsystems, due to cavity-induced noncanonical conditions. Furthermore, we explore the temperature dependency of light–matter quantum entanglement, which emerges for the ground state but is quickly lost already in the deep cryogenic regime. This is in contrast to predictions from the Jaynes–Cummings model, which is the standard starting point to model collective strong-coupling chemistry phenomenologically. Moreover, we find that the fluctuations of matter remain modified by the quantum nature of the thermal and vacuum-field fluctuations for significant temperatures, e.g., at ambient conditions. These observations (loss of entanglement and coupling to quantum fluctuations) have implications for the understanding and control of polaritonic chemistry and materials science, since a semiclassical theoretical description of light–matter interaction becomes reasonable, but the typical (classical) canonical equilibrium assumption for the nuclear subsystem remains violated. This opens the door for quantum fluctuation-induced stochastic resonance phenomena under vibrational strong coupling, which have been suggested as a plausible theoretical mechanism to explain the experimentally observed resonance phenomena in the absence of periodic driving that has not yet been fully understood.

## 1 Introduction

Strong coupling of quantum light and matter via optical cavities has become a rapidly developing technique, which has made an outstanding impact across scientific disciplines over the last few years. For example, exciton-polariton condensates have attractive features for quantum computing^1^ or cavity magnon polariton systems are promising candidates for quantum information processing with long spin coherence times.^2^ Furthermore, modifications of the transition temperature of superconductors were predicted^3−5^ and measured,^6^ and novel optical devices for wavefront engineering and subwavelength focusing became feasible.^7^ Furthermore, the cavity-induced stabilization of the ferroelectric phase in SrTiO_3_ or the magnetic control of proximate spin liquid α-RuCl_3_ have been proposed.^8,9^ Large scientific attention was also created in the chemistry community due to successful inhibition,^10^ steering,^11^ and enhancing^12^ of molecular reaction rates under vibrational strong-coupling conditions.

The decisive ingredient of these experiments is that matter couples strongly to the vacuum or a few thermally created photons of a cavity instead of weakly coupling to many photons under external laser driving. In the latter case, only transient (Floquet-type) nonequilibrium states can emerge, which are hard to detect experimentally due to decoherence, dissipation, and heating effects.^13^ For the strongly coupled cavity–matter system, however, robust thermal equilibrium states of light and matter emerge, which are of significant importance for the physics under investigation (polaritonic states and polaritonic quantum matter).^14^ The theoretical description of quantized light and matter under strong coupling conditions is a notoriously hard problem to tackle as it a priori requires a quantum electrodynamics (QED) description in full thermal equilibrium. To bypass this complexity, simplified models are used predominantly.^15−17^ Many of these models have been devised in quantum optics (e.g., Jaynes– Cummings) and are designed to model photon properties accurately^18^ but at the same time strongly reduce the complexity of the matter subsystem, i.e., the detailed properties of the matter subsystem are assumed irrelevant except for their influence on the light field. This simplification allows us to determine collective scaling effects of large molecular ensembles (e.g., Tavis–Cummings model^17,19−21^). Only recently, the reverse question, i.e., how the strongly coupled photons influence matter properties, has become the focus of intensive research in polaritonic or QED chemistry and materials science.^16,20,22,23^ However, the details of the photon field and an accurate description of the coupled thermal equilibrium are commonly assumed to be irrelevant in this matter-driven perspective, and thus, *T* = 0 is commonly assumed. Yet all of the above specialized viewpoints seem to be insufficient to explain certain experimental observations, such as the resonance condition for suppressing chemical reactions via strong coupling^10,24^ or how strong coupling can influence complex aggregation processes of molecule–metal complexes.^25^

Here, we try to unify these specialized viewpoints based on rigorous theoretical ground, i.e., based on the stationary solution of the exact quantum Liouville equation in the nonrelativistic Pauli–Fierz limit of QED. We then deduce fundamental properties from a paradigmatic molecular test system (HD^+^) considering the full (chemical) complexity, i.e., by having light and matter treated as fully quantized and also including the coupling to an external heat bath. In particular, we will address the following questions: how does the temperature of the total ensemble translate to the individual subsystems? A common simplification is to assume that the effective temperature of the subsystems is equivalent to the temperature of the total ensemble.^26,27^ How are the quantum and thermal fluctuations of the subsystems related? Again, a common simplification is to assume that the fluctuations of the subsystems remain unaffected and can be replaced by the fluctuations of the uncoupled systems. Finally, are light and matter quantum-entangled, and what happens to the entanglement when we increase the temperature? While it is commonly accepted that quantum entanglement should be lost with increasing temperature, a detailed quantification for realistic systems is usually not available. Indeed, the viewpoint of collective “supermolecules”^28−30^ (formed by light and matter at ambient conditions) seems to be contradictory, which is a widely spread concept within the polaritonic community. In this context also, the question of how to define the thermal state and quantum statistics of a collectively coupled ensemble of molecules will become important. Particularly interesting is the fact that we will not focus on the electronic energy range, for which the common quantum-optical models have been designed, but investigate the low-energy ro-vibrational regime instead, which is predominantly affected by temperature. Usually the ro-vibrational degrees of freedom are only considered as decoherence channels for electronic excitations, and their detailed quantum-mechanical nature is not investigated for potential quantum-technological applications. Indeed, molecular systems, in principle, allow us to go beyond simple qubit representations^31,32^ where decoherence sources can be mitigated/controlled by the specific molecular composition.^32−37^ Our results suggest that strongly coupled molecule–cavity systems can possess distillable quantum entanglement in the ground state at ultralow temperatures, and hence, such systems provide a potential platform for the development and implementation of future quantum technologies. Furthermore, for higher temperatures, where entanglement is quickly lost, nontrivial feedback between light and matter points toward cavity-induced noncanonical mechanisms, which become decisive in the context of polaritonic chemistry and materials science. Finally, we extrapolate our findings to more general situations and provide our perspective of molecules under strong vibrational coupling and at thermal equilibrium. We connect this perspective to novel results obtained in the collective coupling regime.

This work is structured as follows: we first discuss how we theoretically describe the quantized light–matter system in the long-wavelength limit of nonrelativistic QED and show the necessary transformations to make the problem numerically tractable. Furthermore, theoretical issues for strongly coupled systems of mixed-particle statistics in thermal equilibrium are addressed. In a second step, numerically exact thermal equilibrium solutions are presented with a focus on strong coupling-induced temperature modifications, quantum thermal fluctuations of light and matter, as well as (loss of) light–matter entanglement for an ab initio molecular system. The entanglement predictions are then contrasted to predictions from the ubiquitous Jaynes–Cummings model of quantum optics, which serves as a cornerstone for collective models. In a third step, a concise picture of cavity-induced (non)canonical effects is developed, and important implications for cryogenic applications are derived (e.g., quantum computing and superconductivity), as well as under ambient conditions (materials science and polaritonic chemistry). We end this work with a forward look and perspective section that we connect to theoretical results obtained in the collective coupling regime.

## 2 Exact Quantum Canonical Equilibrium Solution for HD^+^ Molecule in a Cavity

### 2.1 Hamiltonian Representation

In the following, we rely on the nonrelativistic QED Pauli–Fierz (PF) Hamiltonian in dipole approximation for the fundamental description of the light–matter interaction within a cavity tuned to the infrared or optical regime.^38−40,40−44^ The resulting Hamiltonian assumes the following form in the Coulomb gauge^45^
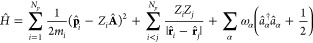
1where *N*_*p*_ is the number of (Fermionic or bosonic) massive particles, i.e., electrons and effective nuclei that constitute the molecules inside the cavity, with *m*_*i*_ and *Z*_*i*_ being the corresponding masses and charges, respectively. For each particle we denote the conjugate self-adjoint momentum and position operators as  and , respectively. The photonic environment is defined in terms of modes α with corresponding frequency ω_α_, linear polarization direction **ε**_α_, and coupling strength (effective mode volume) λ_α_. Here  is the usual bosonic creation and  the annihilation operator for mode α. The quantized transverse vector potential is then given as
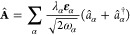
2We have neglected explicitly spin-dependent terms, such as Zeeman and spin–orbit coupling terms, here. The spins of the massive particles become important only for determining the symmetry of the eigenfunctions, i.e., Fermionic antisymmetry and bosonic symmetry under exchange of spin-space coordinates.

Before we continue with necessary restrictions to make the eigenvalue problem posed by eq 1 numerically tractable, we mention an immediate consequence that emerges in comparison with the standard model nomenclature, which distinguishes different coupling regimes of light and matter. Following ref (16), the *weak* coupling regime is dominated by cavity losses over the energy exchange between light and matter (Purcell regime), whereas *strong* coupling refers to the opposite regime, where Rabi oscillations (splitting) emerge. The hybrid light–matter system may also enter the *ultrastrong* coupling regime, which is commonly identified by relating the Rabi splitting Ω to the cavity frequency ω_α_, i.e., it usually starts at Ω/ω_α_ ≈ 0.1. In the ultrastrong coupling regime, counter-rotating terms start to become relevant, which imply modifications of the ground state of the model. Typically, standard models in combination with geometrical restrictions of the cavity (e.g., frequency and mode volume) suggest that strong and ultrastrong coupling conditions can almost exclusively be reached by collective coupling of a large ensemble of molecules, effectively leaving the single molecules unaffected.^16^ While these definitions seem appropriate for atomic systems and electronic strong coupling, in the case of ro-vibrational strong coupling in molecule experiments (see, e.g., ref (20)), recent theoretical results (see, e.g., refs (46) and (47)) suggest that for collectively coupled ensembles, we also find single-molecule (local) strong coupling. We have this case in mind when we consider the HD^+^ coupled to an effective cavity mode. Further localization effects within optical cavities were also reported independently (e.g., in refs (48) and (49)). For this reason and since we can account neither for the above losses of the cavity nor for molecular ensembles, when trying to solve the PF Hamiltonian in eq 1 exactly, we will subsequently use a different definition of strong coupling. Throughout this work **strong coupling** indicates that light and matter hybridizes for a single molecule, i.e., a vacuum Rabi splitting occurs on a single molecular level in the absence of any cavity losses (e.g., local strong polarization of an impurity due to a surrounding collectively coupled ensemble^46,50^). Notice that the numerically exact solution of eq 1 automatically accounts for cavity-induced modifications of the ground state for arbitrarily small λ_α_ > 0.

However, considering that a single molecule is not enough to solve the PF Hamiltonian exactly on a computer, a few more simplifications are necessary: first, we restrict to one effective mode α of the cavity. As a next step, we restrict to three particles, i.e., *N*_*p*_ = 3. This allows us to treat, e.g., a helium atom, an H_2_^+^, or an HD^+^ molecule.^51^ Here, we choose an HD^+^ molecule, that is, a positively charged molecule with one proton, one deuteron, and one electron. While solving more than three quantized particles exactly is possible nowadays with computational power for pure matter systems (e.g., H_2_ in ref (52).), this still seems out of reach if the molecule is strongly coupling to a quantized cavity mode, since the usual tricks with separating off rotational degrees of freedom do not apply anymore, i.e., the single mode effectively increases the dimensionality of the problem and not only by one. Therefore, additional approximations become necessary for more complex molecules, as, for example, done by exchange correlation functionals in QEDFT^41,53−56^ or by QED coupled cluster methods.^57−59^ Having numerically exact eigenvalues and eigenstates available for HD^+^ will subsequently allow us to investigate exact thermodynamic equilibrium properties and light–matter entanglement under ro-vibrational strong coupling. For this purpose, we briefly recapitulate the key technical ingredients of our problem-adapted numerical approach as they become essential for the subsequent discussions.

To achieve a numerically tractable form of our quantized three-body problem coupled to one quantized cavity-photon mode in the long-wavelength limit, the corresponding nonrelativistic Pauli–Fierz Hamiltonian has to be expressed in center-of-mass (COM)  and relative coordinates **r**_*ci*_ = **r**_*i*_ – **R**_*c*_. Moreover, a relative velocity form of the Hamiltonian becomes important,^51^ which is obtained from a unitary Power–Zienau–Woolley transformation
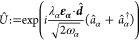
3where the relative dipole operator was introduced as
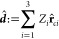
4Next, we perform a canonical commutator-preserving substitution *S* of the photon operators, i.e.,  and , resulting in^51^
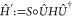
5
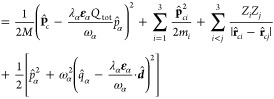
6Here, *Q*_tot_: = ∑_*i*_^3^*Z*_*i*_ is the total charge and *M*: = ∑_*i*_^3^*m*_*i*_ is the total mass of the three-particle system. We note that the canonical variable  and its conjugate momentum  correspond to the displacement field and we have thus mixed the original light and matter degrees of freedom of the Coulomb gauge.^60,61^ Thus, physical observables of the photon field, e.g., the transverse electric field fluctuations, can depend on the displacement, COM, and relative coordinates (see, e.g., eq 22). The resulting stationary eigenvalue problem can be solved numerically and exactly using the wave function ansatz
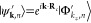
7where we have chosen the cavity mode polarized along *z*, wave vectors **k**, and quantum numbers *n*. The solution can be achieved by a smart choice of a spherical–cylindrical coordinate system, where angular integrals are treated analytically and radial integrals are treated numerically by using Gauss–Laguerre quadrature.^51,62,63^ From the choice of our gauge, an interesting property of the Hamiltonian becomes immediately evident for charged molecules with *Q*_tot_ ≠ 0, e.g., HD^+^. For those molecules, the COM motion directly couples to the displacement field of the cavity,^51^ which will add additional numerical complexity to our subsequent numerical treatment in thermal equilibrium. Finally, we would like to stress that in our approach, all quantized matter degrees of freedom (i.e., nuclei and electrons) are strongly coupled to the cavity mode. In particular, the explicit coupling to the nuclear sector can become decisive for the physically accurate description of (ro)-vibrational strong coupling.^64,65^

### 2.2 Thermal Equilibrium in Polaritonic Systems

The rigorous quantum statistical treatment of a hybrid light–matter system poses interesting theoretical questions since it contains bosonic and Fermionic degrees of freedom that are strongly mixed (we note here that the nuclear degrees of freedom can be both Fermionic or bosonic, depending on the effective spin of the nuclei). In the general case, the canonical equilibrium density operator ρ̂ is a stationary solution of the quantum Liouville equation
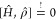
8subject to the constraints of constant particle number, volume, and temperature. The canonical density operator takes the following general form at temperature *T*
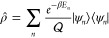
9assuming canonical typicality for the entire system, which shall be weakly coupled to a thermal bath.^66^ The *N*_*p*_-particle eigenenergies are defined as *E*_*n*_ with the corresponding eigenstates |ψ_*n*_⟩. The canonical partition function is given by  with β = 1/(*k*_B_*T*). In the traditional uncoupled case, i.e., for λ_α_ = 0, where the *N*_*p*_ fundamental particles in eq 1 could, for example, form *N* spatially distinct molecules (assuming dilute limit), we can simplify the problem by means of statistical physics. Hence, we can treat these *N* molecular entities either as effective bosons or Fermions, i.e., we can occupy the new quasi-particle states according to a Fermionic or bosonic statistics. In more detail, we can thermally populate the corresponding *N*-particle states  for effective Fermions or  for effective bosons with . Here, we have introduced the single-molecule eigenenergies *E*_*n*_^(1)^ and eigenstates |ψ_*n*_^(1)^⟩. For the uncoupled case λ_α_ = 0, the bare photon modes α obey the usual Bose–Einstein distribution. Consequently, the thermal density matrix operator of eq 9 would just be a tensor product of the thermal density matrix of the (noninteracting) molecules and the uncoupled photon modes.

In the strong coupling case λ_α_ > 0, things become complicated. In this case, this simple tensor product ansatz might, however, be no longer sufficient since the matter and photon degrees of freedom can strongly mix and we *a priori* lose a clear entity to treat statistically (e.g., spatially separated molecules). Indeed, the assumption (sometimes employed in polaritonic chemistry) that light and matter can form a coherent “supermolecule” inside a cavity^28−30^ would suggest that we should treat the complete ensemble of molecules plus cavity as a single quantum entity.^28−30,67,68^ If this were the case also for higher temperatures, we would have a macroscopic quantum state under ambient conditions with potential quantum entanglement between the cavity and the ensemble of molecules, which seems rather implausible. Moreover, in this case, the fundamental quantum statistics of the individual *N*_*p*_ particles as used in eq 9 might become dominant and we need to consider the individual particles completely delocalized over macroscopic distances at ambient conditions. In our specific case, we would have a strong coupling between the different protons (Fermionic), deuterons (bosonic), and electrons (Fermionic) with the quantized light field. While the rigorous quantum treatment of such an ensemble of molecules is numerically not feasible, we can investigate thermal quantum properties (e.g., light–matter entanglement) for the simplest case, *N* = 1, i.e., we just have a single HD^+^ molecule strongly coupled to the cavity. In this case, we will have access to the exact thermal density matrix of eq 9 since we can calculate the lowest-lying (ro-vibrational) eigenstates of eq 7. The numerical details of our approach are described in the Supporting Information of ref (51) as well as in Section S1 of the Supporting Information of this work with focus on the thermal quantum ensembles.

A few remarks: it is important to contrast the above notion of chemical systems being quantum-coherently coupled with other types of effective quantum models for excitations, e.g., exciton-polaritons. In these situations, it is not the wave function of the ensemble of molecules that is being considered but the excitation’s quasi-particle instead, i.e., it is merely the quantized excitations that are being transferred between fixed molecular structures. We further note that in the case of variable particle numbers, the statistical grandcanonical ensemble should represent different realizations of *N*_*p*_ particles coupled to a cavity as opposed to an indefinite (Fock-space) number of particles coupled to a single cavity. However, the grandcanonical treatment will not be discussed further in this work.

## 3 Exact Quantum Properties for Vibrational Strong Coupling at Finite Temperature

Having the numerically exact thermal equilibrium density operator available for an ab initio representation of a real molecule under vibrational strong coupling conditions enables one to approach the questions raised in the introduction. In the following, we will see how the strong light–matter coupling condition induces finite temperatures for the matter and light subsystems despite keeping the total system temperature at 0 K, and how the subsystem temperatures approach the canonical temperature when we couple the cavity–molecule system to an external heat bath. Next, we investigate the effect of the hybridization between light and matter on thermal and quantum fluctuations. Finally, we discuss quantum entanglement between light and matter at cryogenic temperatures yet show how increasing the temperature destroys quantum entanglement, contrary to predictions from the ubiquitous Jaynes–Cummings model.

### 3.1 Temperature under Strong Coupling Conditions

Having numerically exact canonical ensemble densities available at temperature *T*, it is interesting to investigate how the strong coupling conditions affect the separate molecular and photon subsystem temperatures. As discussed above, the presence of the strongly coupled cavity mode breaks the common weak coupling assumption for the matter subsystem, which will lead to a noncanonical thermal subsystem density matrix operator. Notice that such noncanonical effects are straightforward and expected from a theoretical perspective.^69−71^ However, they are commonly discarded in polaritonic chemistry^17,20,21^ and, to our knowledge, have not yet been investigated at all from first principles. In the following, we quantify the cavity-induced temperature effects on the matter and light subsystem levels for strong vibrational strong coupling.

For this purpose, we introduce a natural definition of subsystem temperatures τ in terms of the reduced density matrix (RDM) formalism, which will provide access to subsystem equilibrium properties, given that the full light–matter system is in canonical equilibrium at temperature *T*. Naturally, the definition of a subsystem temperature τ_*W*_ for a strongly coupled subsystem *W* involves some ambiguities, as we will see, except for the weak coupling limit λ_α_ → 0, where one should recover canonical properties for the subsystem *W*, i.e., τ_*W*_ → *T*. We also note the connection to quantum embedding schemes such as subsystem density functional theory^72^ or density matrix embedding theory.^73,74^ Let the RDM operator of ρ̂ given in eq 9 be defined by
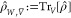
10for a bipartite partitioning of the full polaritonic system *W* ⊗ *V*. The bar indicates the traced out vector space *V*. It is straightforward to show that  remains a self-adjoint operator with , due to the normalization of the ensemble density matrix operator  by the canonical partition function . Because  is self-adjoint on the Hilbert space *W*, we have a unique diagonal representation
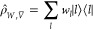
11This always allows us to define, for an arbitrary (!) temperature τ_arb_, a self-adjoint operator for which  represents a canonical ensemble. We can do so by choosing *E*_*l*_^arb^ such that
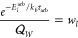
12which leads to  with . So, to find a physically reasonable definition of a subsystem temperature, we need to fix the subsystem Hamiltonian . In the case of coupled light–matter systems, this can be done naturally by taking λ = 0 in eq 6 and considering the decoupled light and matter Hamiltonian. In this case we can further subdivide the matter Hamiltonian in COM and the relative matter system, i.e., we use the notation *W* ∈ {pt, COM, m} for the different subsystems. Using the corresponding subsystem Hamiltonians we can then determine
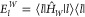
13and then numerically get the corresponding subsystem temperature τ_*W*_ by the fitting
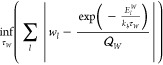
14We expect this choice to be reasonable for moderately interacting subsystems, which remain close to an equilibrium at τ_*W*_ ≈ *T*, since for any noninteracting subsystem *W* and *V* (e.g., photon mode and matter at λ_α_ = 0), the RDM operators equal the subsystem canonical density matrix operators , i.e.,  with *T* = τ, by construction. This automatically implies that our subsystem temperature definition is unlikely to be a reasonable concept anymore for strongly interacting subsystems. In that case, there is no reason to expect an exponential fitting for τ_*W*_ to capture the relevant physics and the noncanonical contributions would be dominating. In practice, we expect that the quality of approximating the full system with a canonical RDM at fitted temperature τ is hard to determine and will depend on multiple aspects such as the molecular system, cavity parameters, and the observable of interest. In particular, a high goodness of fit would still not guarantee that noncanonical features are irrelevant for a certain observable of interest.^75^ Notice, however, that usually one does not have access to τ and thus simply assumes the existence of weakly interacting subsystems of interest by setting τ = *T*, e.g., when thermostatting molecular dynamics simulations, which considerably reduces the computational complexity of the problem.^26,27^ This cannot necessarily be imposed under strong vibrational coupling conditions, as we show subsequently.

Let us first consider a simple COM subsystem. For the temperature of the COM motion, one immediately finds
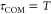
15because the eigenfunctions of our fully coupled HD^+^ Hamiltonian given in eq 7 ensures that the full Hamiltonian and ensemble density matrix operator are block-diagonal with respect to the quantum numbers *k*. Therefore, the partial trace operation acting on the relative matter and photonic degrees of freedom reduces each block to one dimension. Consequently, both reduced matrices are diagonal, which trivially obey . This implies that the COM dynamics obey strict canonical equilibrium within the long-wavelength limit of the Pauli–Fierz theory. This is a nice consistency between the classical idea of the temperature of a gas, which assumes a certain distribution of velocities of particles, and the quantum-mechanical treatment.

**Figure 1:**
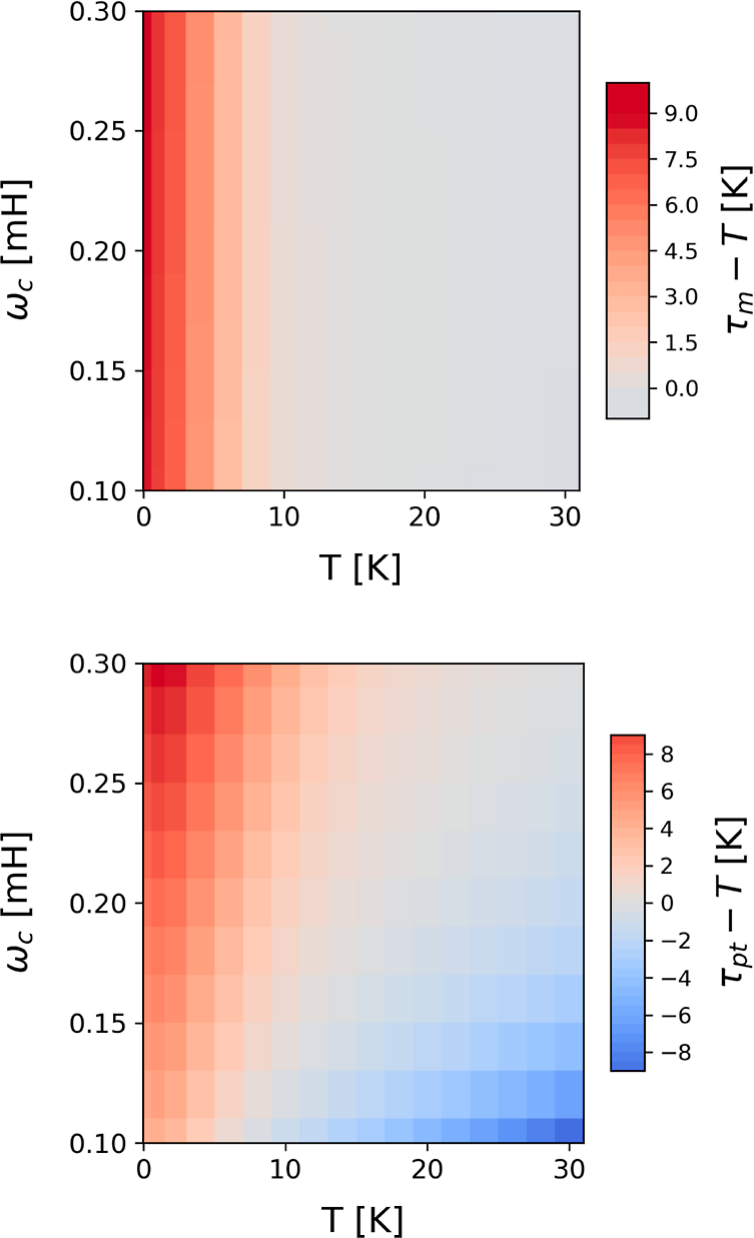
Top: heating (red) and cooling (blue) effects for the matter subsystem temperatures’ difference with respect to the total system temperature τ_m_ – *T* emerging from cavity-induced noncanonical conditions under vibrational strong coupling for λ_α_ = 0.005 at different *ℏ*ω_α_ and for different total system temperatures *T*. Bottom: the same analysis is used for cavity-induced modifications of the photon mode temperature difference τ_pt_ – *T* under vibrational strong coupling.

However, things change fundamentally for the relative matter temperature τ_m_(*T*, λ_α_, ω_α_) and photon temperatures τ_pt_(*T*, λ_α_, ω_α_), as displayed in Figure 1 for ro-vibrational strong coupling with λ = 0.005 [a.u.] for frequencies close to the first ro-vibrational excitation of HD^+^ at ω = 5.4 meV. Notice that the aforementioned block-diagonal nature of the full ensemble density matrix significantly simplifies the numerics of those calculations because the partial trace operation acting on the COM subsystem then effectively reduces to the trace operator summing over *k*_*z*_. Our temperature definitions already indicate that the strong light–matter coupling induces different heating as well as cooling effects on the subsystems, which depend on the fixed temperature *T* and coupling λ_α_ and partially on the cavity mode frequency ω_α_. In more detail, we observe two different regimes for the matter temperature τ_m_. It converges to a finite minimal matter temperature for *T* ≲ *T*_0_ = 10 K. This lower bound for the matter temperature τ_m_(*T* → 0, λ_α_ > 0, ω_α_) > 0 and the corresponding transition temperature *T*_0_ strongly depends on the chosen light–matter coupling λ_α_, and virtually no dependency on the chosen resonance frequency ω_α_ was observed. As we will see also in the following sections, at approximately *T*_0_, not only does the matter subsystem temperature start to deviate strongly from the externally defined (canonical) temperature but also other important properties of the coupled light–matter system change their character. The transition temperature *T*_0_ is therefore a characteristic quantity of the coupled HD^+^ system. For *T*_0_ ≲ *T*, we find that τ_m_ ≲ *T*, i.e., it almost corresponds to the temperature of the total system with a slight cooling involved. For the temperature of the strongly coupled photon mode τ_pt_, we find a different behavior. Still, at low *T* ≲ *T*_0_, there is a clear heating observed, i.e., τ_pt_ > *T*. However, this turns into a significant cooling τ_pt_ < *T* for larger *T*. In contrast to the relative matter subsystem, the magnitude of the heating and cooling regimes strongly depends on the chosen cavity frequency ω_α_. The qualitative difference of τ_pt_ and τ_m_ is not surprising, since HD^+^ is a charged molecule. Therefore, the thermal COM motion *k*_*z*_ ≠ 0 along the polarization **ε**_α_∥***k***_*z*_ will significantly affect the photon field, i.e. the thermal center of charge motion is formally equivalent to the pumping of the cavity with external currents. A priori this “temperature pumping” effect will directly increase the photon number and thus affect τ_pt_ but much less so (i.e., only indirectly) the relative molecular system.

We want to highlight that at ultralow temperatures *T* ≈ 0, the vibrational strong coupling seems to induce a noncanonical condition for the subsystems, which can be regarded as a significant heating, i.e., the absence of 0 K for matter and light. This finding may be relevant for the future interpretation of experimental data in the low cryogenic regime, e.g., for modifications of the critical temperature of cavity-assisted superconductivity^6^ and other polaritonic phenomena (condensates) at low *T*. Furthermore, recent experimental evidence for ro-vibrationally strong coupled 1T-TaS_2_ (published shortly after the initial version of this manuscript) indeed suggests that cavity-induced heating effects can influence material properties.^76^ In more detail, Jarc et al. found that the critical temperature associated with the metal-to-insulator transition is lowered off-resonantly.^76^ This experimental data resembles what one would expect qualitatively from Figure 1, i.e., it indicates the practical relevance of the observed subsystem temperatures for light and matter under ro-vibrational strong coupling conditions.

### 3.2 Cavity-Modified Thermal Fluctuations

In a next step, we investigate how the strong ro-vibrational coupling affects the (vacuum) field mode and matter fluctuations in thermal equilibrium. Reaching a detailed understanding of cavity-modified fluctuations is not only of theoretical interest but it is also of fundamental importance for the emerging fields of polaritonic chemistry and materials science, where modified fluctuations would call for an adaptation of usual molecular dynamics simulations^24^ with corresponding noncanonical rate theories.^24,77^ For example, changing the dynamics (fluctuations) of matter in a (cavity)-frequency selective manner under thermal equilibrium conditions opens new pathways to steer and control chemical reactions.^11^

For this reason, we subsequently investigate the exact field and matter dipole fluctuations accessible for our HD^+^ molecule under ro-vibrational strong coupling. As previously stated, the strongly coupled HD^+^ molecule is diagonalized in the COM-relative length gauge which follows from the transformation given in eq 3. Consequently, to obtain physically meaningful results, we also need to transform the usual Coulomb-gauged observables to our gauge choice. That is, when evaluating the respective physical observables *Ô* defined in (the velocity form of the) Coulomb gauge (see eq 1), we consider  instead, where the pt-coordinate transformation  preserves canonical commutation relations.

We find the transformed vector potential , the displacement field , and the transverse electric field  operators, polarized along the polarization axis of the cavity *z* as
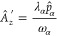
16
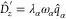
17
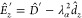
18

Notice that the physical transverse electric field operator corresponds to the displacement field operator in the standard velocity form of the Hamiltonian operator given in eq 1, i.e., . However, our specific gauge choice introduced the dependency on the relative dipole operator, as given in eq 18.

The conservation of the parity symmetry *P* for the Hamiltonian operator as well as for our COM-relative gauge has interesting consequences for the fully quantized system. From the Hamiltonian invariance under , a zero transversal field and zero dipole condition follow
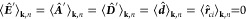
19This implies that we need to break the parity symmetry of the Hamiltonian in order to have a finite molecular dipole, e.g., by fixing the nuclei on a Born–Oppenheimer surface. In this way, we choose a specific realization of the otherwise symmetric possibilities of free space.^78^ In practice, the choice of which possibility is realized is then governed by the local environment. This has interesting consequences for a potential “supermolecule” of a quantum-coherent ensemble of molecules, as we will discuss later. Consequently, photon field and matter dipole fluctuations of the form  can entirely be described by
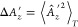
20
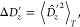
21
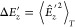
22
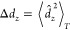
23Notice that eq 19 allows us to disentangle thermal electric field fluctuations Δ*E*_*z*_^′^ in terms of dipole Δ*d*_*z*_ and displacement field fluctuations Δ*D*_*z*_^′^ as well as their respective quantum correlations following from . The magnitude of these gauge-dependent (!) quantum correlations is a priori of no physical interest. However, it becomes a relevant quantity for the future development of approximations in theoretical models or for simulation methods under cavity-modified thermal equilibrium conditions (e.g., in terms of open quantum systems^24^ or within semiclassical cavity Born–Oppenheimer molecular dynamics^24,64^). While in principle different gauge choices are equivalent, in practice, it can become decisive for the numerical representation. For example, the length form (coupling of the field to the matter dipole operator) is usually computationally favorable for molecules, whereas in solids, typically the velocity form (the field couples to the momentum operators) is commonly applied, which is better suited for periodic boundary conditions.

**Figure 2:**
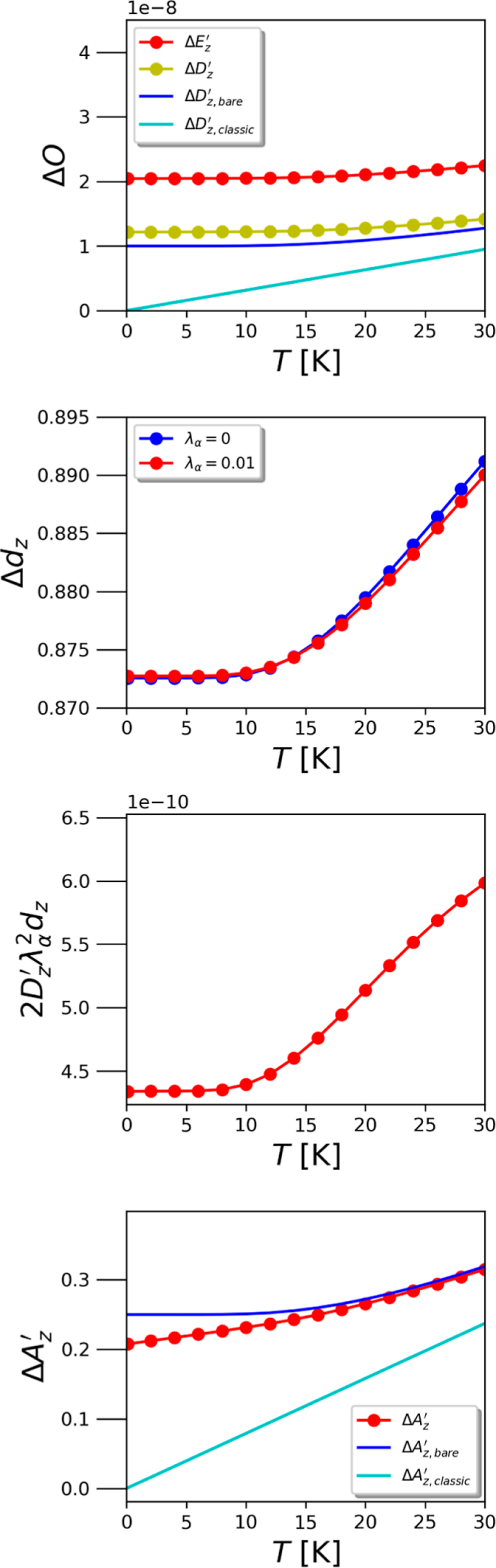
From the top to the bottom: thermal quantum fluctuations for electric Δ*E*_*z*_^′^ and displacement field Δ*D*_*z*_^′^, dipole Δ*d*_*z*_, dipole–displacement correlations 2*D*_*z*_^′^λ_α_^2^*d*_*z*_, and vector potential Δ*A*_*z*_^′^ operators. Comparing to the uncoupled fluctuations (λ = 0) reveals two different fluctuation regimes below and above *T*^0^ ≈ 15 K for a coupling strength of λ = 0.01 and the cavity tuned on the first ro-vibrational excitation of HD^+^.

When tuning the cavity on resonance with the first ro-vibrational excitation of HD^+^, we find the temperature dependency of the fluctuations as shown in Figure 2 for λ_α_ = 0.01. When evaluating the ensemble averages of the operators given in eqs 20–23, we observe a significant increase (shift) in the transverse electric field fluctuations Δ*E*_*z*_^′^ compared with thermal vacuum fluctuation  of a bare cavity mode due to the strong coupling with matter. The displayed analytical electric/displacement field fluctuations of a bare cavity mode can be calculated analytically as
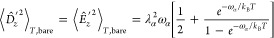
24which converges to
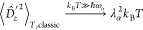
25in the classical limit for *k*_B_*T* ≫ *ℏ*ω_α_. While the dressed electric field fluctuation overall is shifted to higher values, the temperature dependency remains more or less preserved with respect to the thermal quantum fluctuations of a bare cavity mode. For the thermal matter fluctuations of the coupled dipole operator, i.e., for Δ*d*_*z*_, we find a slight suppression at temperatures *T* < *T*^0^ ≈ 15 K with λ_α_ = 0.01, followed by an increase in the fluctuations at higher temperatures, which indicates the transition to a different regime of physics at a temperature *T*^0^, which is in agreement with the previous observations for the subsystem temperatures. Similarly, the gauge-dependent light–matter quantum correlations of the form  change from a slight increase to a small suppression. However, overall they remain negligibly small, i.e., 2 orders of magnitude smaller than the physically relevant transverse electric field fluctuations. Consequently, quantum correlations between the dressed displacement field and the matter dipole could safely be neglected, which opens room for efficient approximations to investigate more involved systems. In contrast to the increase in the transverse electric fluctuations, our simulation shows that the thermal fluctuations of the vector potential Δ*A*_*z*_^′^ are suppressed most significantly at low temperatures *T* ≲ *T*^0^, compared with a bare cavity mode
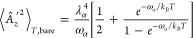
26with its classical counterpart for *k*_B_*T* ≫ *ℏ*ω_α_
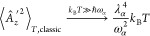
27While beyond *T*^0^, the quantum harmonic oscillator solution is quickly approached.

Overall, the theoretically predicted suppression of matter fluctuations Δ*d*_*z*_ at temperatures far beyond *T*^0^ confirm from first principles that the equilibrium dynamics of matter can indeed be substantially modified by ro-vibrational strong coupling to the quantized cavity modes, as proposed in ref (24) (before reaching the classical limit *k*_B_*T* ≫ *ℏ*ω_α_). This observation has a potential impact on the future development of polaritonic reaction rate theories and noncanonical equilibrium simulation methods, which are crucial for the design of novel cavity-mediated reaction processes and for cavity-mediated modifications of the equilibrium ground state in quantum materials. Aside from the significantly modified matter dynamics at high temperatures, the discovered transition to a different fluctuation regime for cryogenic temperatures *T* < *T*^0^ raises the question of the underlying physical mechanism, which we will discuss in the following.

### 3.3 Cavity-Induced Light–Matter Entanglement at Finite Temperature

Apart from identifying cavity-mediated heating/cooling and correlating thermal fluctuations between light and matter, our numerically exact solution of HD^+^ in a cavity also allows us to assess the “quantumness”, i.e., the quantum entanglement of the light and matter, at finite temperatures. Entanglement between light and matter would make strongly coupled molecule–cavity systems for ro-vibrational frequencies interesting for potential applications in quantum information processing. This would be specifically true if this entanglement would be thermally stable for sizable temperatures. We further note that to the best of our knowledge, this is the first study that computes light–matter entanglement for an ab initio molecular system in a cavity, i.e., one does not rely on a model Hamiltonian that treats the matter degrees of freedom in a strongly simplified manner.

To investigate this question, we will determine the temperature-dependent light–matter entanglement under ro-vibrational strong coupling. For this purpose, we rely on the logarithmic negativity
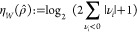
28which is a computationally efficient (i.e., not *NP*-hard) bipartite entanglement measure applicable to mixed states of distinguishable particles.^79−83^ The negative eigenvalues ν_*i*_ are calculated from the partial transpose of the ensemble density operator  with respect to the chosen subsystem *W* in a bipartite partitioning. Fortunately, the necessary distinguishability criterion is certainly fulfilled for our dressed HD^+^ molecule since its three Fermionic constituents are different (electron, proton, and deuteron) and couple to one bosonic cavity mode only. The logarithmic negativity entanglement measure serves as an upper bound for the distillable entanglement.^83^ However, a zero logarithmic negativity does not imply that the bipartite subsystems are not entangled, since a bound entangled state cannot be detected.^84^ This has particularly interesting implications for our charged COM motion, which directly couples to the photon field. Indeed, the COM motion in a cavity provides a nice example of a bound entangled state with respect to the rest of the system. In more detail, we find
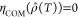
29because of , which uses the fact that ρ̂ is block-diagonal with respect to ***k***. However, at the same time, the COM subsystem is not separable provided that . Under these circumstances, the charged COM motion along *z* couples to the photon field, i.e., both factors in the exact eigenfunction given in eq 7 depend on *k*_*z*_. Consequently, the COM partition forms a bound entangled pair with the rest of our system.

**Figure 3:**
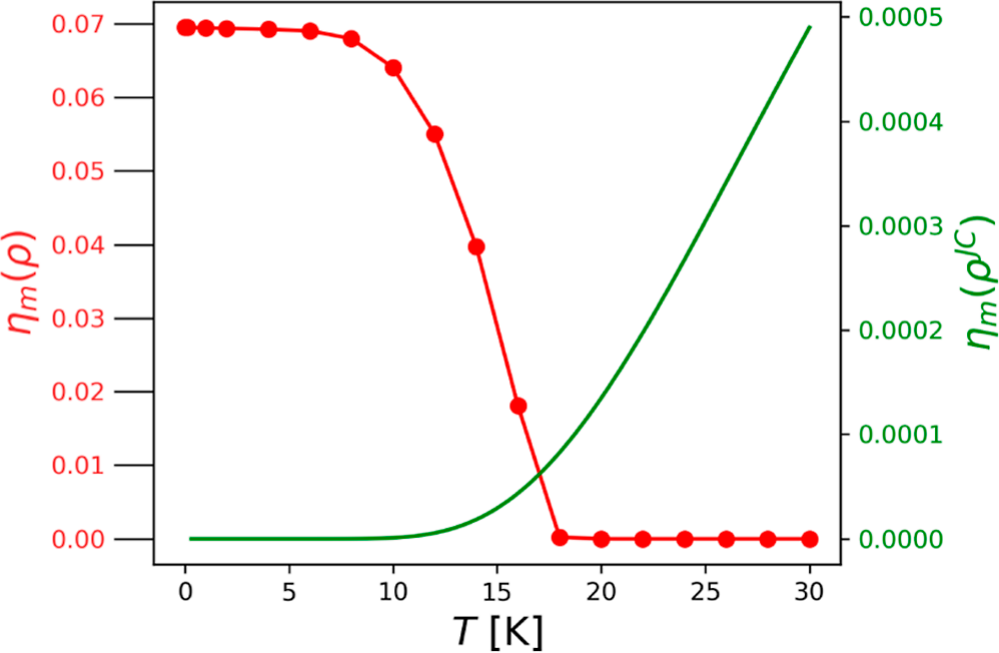
Logarithmic negativity measure for the entanglement of light and matter for a HD^+^ molecule in a cavity under thermal equilibrium conditions at resonant coupling with λ_α_ = 0.005. The exact equilibrium solution of the Pauli–Fierz Hamiltonian (red) shows constant entanglement up to *T* ≈ *T*^0^, which is then quickly destroyed thermally. In contrast, the Jaynes–Cummings model (green) suggests the opposite behavior, i.e., thermal entanglement creation by mixing the bare matter ground state with excited polaritons. Notice that we can only compare the measures qualitatively but not quantitatively. The reason for this is related to the different Hilbert spaces the model and the exact solution are acting on, particularly since the logarithmic negativity is not an asymptotically continuous measure.

Now, let us take a look at the entanglement between light and matter for our dressed HD^+^ molecule. The detailed numerical procedure to determine the logarithmic negativity
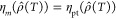
30for our system is given in Section S2 of the Supporting Information. Because we have already discussed the fact that the coupled COM degrees of freedom cannot contribute to the logarithmic negativity, any nonvanishing value of η_*m*_ can be attributed to entanglement between the relative matter subsystem and the photon field. In Figure 3, the numerically exact η_*m*_ is displayed (in red) with respect to the temperature *T*, where we have set the coupling to λ_α_ = 0.005 and tuned the cavity on resonance with the first ro-vibrational excitation. We find significant, almost constant, light–matter entanglement η_*m*_ between 0 and *T*^0^ ≈ 10 K, which then quickly drops for higher temperatures and remains zero for temperatures beyond 18 K. Consequently, the different physical regime for *T* > *T*^0^ seems to be a consequence of the thermal extinction of the entanglement between light and matter. This indicates that a semiclassical description for the coupling of light and matter might cover most relevant aspects for temperatures beyond *T*^0^. In contrast, the observation of a nonzero logarithmic negativity measure indicates that our hybridized thermal state (dominated by the coupled light–matter ground state) would in principle be suitable for quantum computing in the cryogenic regime *T* < *T*^0^. However, as soon as the temperature becomes large enough such that a sizable contribution from the excited states is mixed with the ground state, the light–matter entanglement is lost. By increasing the coupling parameter λ_α_, one can, in principle, reach entanglement at slightly higher temperatures. However, overall, it will be limited by the thermal population of the lowest ro-vibrational excitation, i.e., our simulations confirm the expectation that cavity-induced light–matter entanglement for vibrational strong coupling can only be achieved under thermal equilibrium conditions at ultralow temperatures. This effect is a direct consequence of the hybridization of light and matter in the ground state. Neglecting this delicate aspect of cavity-induced ground state modifications, as is commonly done in models applied to (collective) vibrational strong coupling situations,^18^ can lead to qualitatively and quantitatively different results. Particularly, the Jaynes–Cummings model serves as the de facto standard when interpreting vibrational strong coupling situations, since it allows a simple scaling to large ensemble sizes (Tavis–Cummings model).^17,20,21^ It is important to remark that there would be more sophisticated models available, which may qualitatively better capture the numerically exact results (e.g., Rabi or Dicke model).^85,86^ However, including counter-rotating and self-interaction terms in the derivation effectively hampers the scaling to large collective ensemble sizes,^64,65,87,88^ and their nontrivial implementation for canonical equilibrium conditions goes beyond the scope of this work. We stress again that in the current setup, the cavity is resonantly coupled to the lowest *ro-vibrational* states, which are also the ones that are thermally populated. So, from a model perspective, there are only a few discrete matter states (see also, a detailed discussion in ref (51)) that are coupled, and hence, a straightforward extension of the usual atomic (electronic excitation) models seems reasonable. Using other models, such as the Holstein–Tavis–Cummings model or the PoPES model, where vibrations are treated rather as a source for decoherence and dephasing,^87,88^ does not seem more appropriate.

In the following, we therefore apply the logarithmic negativity measure to the ubiquitous Jaynes–Cummings model used in the construction of collectively coupled polaritonic systems. One finds (see Section S3 of the Supporting Information)
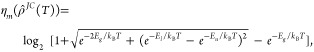
31assuming a cavity tuned on resonance with the bare matter excitation. The Jaynes–Cummings model implies a bare matter ground state and thus automatically leads to
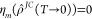
32Indeed, neglecting the hybrid light–matter nature of the ground state introduces a thermal light–matter entanglement, as shown in Figure 3 in green, i.e., the thermal mixing of the eigenstates creates entanglement under equilibrium conditions instead of (correctly) destroying it with increasing temperatures. Consequently, at least from an entanglement perspective, it might be more appropriate to model vibrational strong coupling for sizable temperatures with semiclassical (with respect to the light–matter coupling) methods rather than with Jaynes–Cummings-type approaches.^24,89^ Notice that in our work, we investigate light–matter entanglement under thermal equilibrium conditions, which is in agreement with many experiments on modifications of ground-state chemical reactions by vibrational strong coupling.^90^ In contrast, when preparing the system initially in an excited polaritonic state (e.g., lower or upper polariton), things will change, and the Jaynes–Cummings model may become a reasonable approximation for vibrational strong coupling from the entanglement perspective as well. In such cases, light–matter entanglement can potentially occur at much higher temperatures.^91^ In contrast, the ground state-dominated light–matter entanglement at thermal equilibrium corresponds to a stationary solution of the system (eq 8), which, at sufficiently low temperatures, is long-lived and robust. We note that even including the full continuum of modes of the electromagnetic field, i.e., radiative dissipation, will keep the ground state of the molecule infinitely lived and, thus, a true bound state in the continuum which is completely decoherence-free.^40^

## 4 Discussion, Conclusions, and Outlook

Let us finally collect all of the different results we have obtained from this numerically exact ab initio example of molecular polaritons at finite temperatures. We have coupled the lowest *ro-vibrational* states resonantly with one effective cavity mode. Scanning the frequency of the cavity and the temperature of the thermal bath, we can identify three different regimes:

(i) First, at low cryogenic temperatures *T* < *T*^0^ of the combined system, we find **light-matter entanglement** η(λ_α_) > 0, which arises from cavity-induced modifications of the ground state. Consequently, an accurate theoretical description requires a priori the full quantum treatment of light and matter. This automatically implies cavity-induced **noncanonical quantum dynamics** for the respective subsystems in the absence of external driving. Furthermore, having distillable quantum-entangled states available in the ground state of molecular polaritonic systems may also be of interest for the design of robust entangled states suitable for quantum computing.^32,34,92^ Note also that the heating of the subsystem temperatures due to strong light–matter interaction effectively prevents the subsystems from reaching 0 K, despite approaching the hybridized ground state of the total system at 0 K.
(ii)By increasing the system’s temperature *T*, thermal mixing of eigenstates quickly destroys the quantum entanglement between light and matter at *T* > *T*^0^ even in the strong coupling regime. Consequently, we enter the regime of **correlated light-matter dynamics** (see also Figure S1 of the Supporting Information for IR-to-visible strong coupling regimes). However, the field fluctuations are still governed by quantum laws influencing the matter via strong coupling, even in the absence of light–matter entanglement. We can distinguish two subcases:
(a)At low thermal energies, i.e., *T*^0^ < *T* ≤ *ℏ*ω_α_/*k*_B_, the disentangled field fluctuations are mainly driven by the vacuum fluctuations of the (dressed) ground state of the hybrid light–matter system. Overall, the coupling to matter enhances the fluctuations compared with a bare cavity mode [i.e., in our setup, the coupled transversal electric (vacuum) field fluctuations are doubled].
(b) At moderately higher temperatures *ℏ*ω_α_/*k*_B_ ≲ *T*, thermal mixing of few excited states start to contribute to the field fluctuations. Therefore, a quantum thermal description is still required before reaching the classical thermal limit for *k*_B_*T* ≫ *ℏ*ω_α_.
(iii) In the high temperature limit *k*_B_*T* ≫ *ℏ*ω_α_, the thermal fluctuations of the cavity eventually approach the classical limit, which suggests that we reach **classical canonical nuclear dynamics**(24)

**Figure 4:**
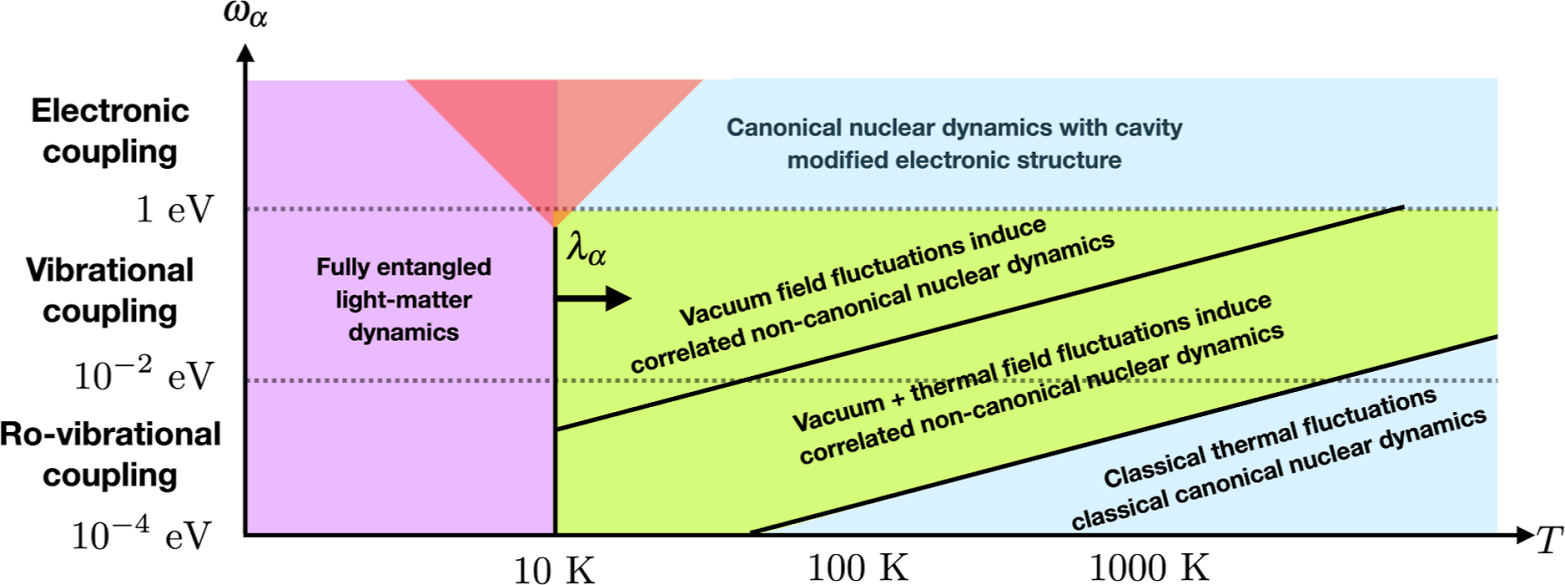
Pictorial sketch of different thermal subsystem regimes emergent under molecular strong coupling conditions for one HD^+^ molecule coupled to a single cavity mode. The red triangle indicates higher coupling frequencies, where no entanglement data were calculated for the HD^+^ setup. Notice that the pictorial sketch changes fundamentally if the hybrid light–matter system were externally driven out of equilibrium. In this case, light–matter entanglement would likely occur at much higher temperatures too.

We have summarized these findings in Figure 4. Next, we would like to comment on what our findings might imply for other physical and chemical setups. After 6 (!) different referees, who unanimously agreed that the results are technically correct, timely, and interesting but who all interpreted the results in very different ways, we think that some more general (interpretative) discussions are worthwhile. We disagree with suggestions that our results are not allowed to be interpreted and should only serve as benchmarks for further approximations. Instead, we at this point warn the reader that we will next make some educated guesses and connect to other results obtained for different physical and chemical situations. We believe that the reader is capable of judging the plausibility of the following arguments.

Let us first discuss in which manner the observed results can be generalized to other molecular systems under ro-vibrational single-molecule strong coupling. Thanks to the exact diagonalization of the corresponding Hamiltonian, the number of initial assumptions and approximations for this specific setup could be reduced to a minimum. Besides the exact coupled eigenstates and thermal density matrix, we also have access to the bare (uncoupled) eigenstates and thermal ensemble. Having the exact bare eigenstates available and due to the resonant coupling, a simple JC modeling suggests itself. However, as can be seen from previous work (see Section S3.2 of the Supporting Information of ref (51)), the JC wave function ansatz for these ro-vibrational states is not as accurate as that for atomic systems (electronic excitations of He) (see Section S3.1 of the Supporting Information of ref (51)). Although this disagreement is enhanced by considering a charged molecular system, we believe that care should be taken when atomic approximations are applied to molecules. Also note that since we couple resonantly to ro-vibrational eigenstates, vibrations are not mere decoherence channels as often implicitly assumed. Considering the three different regimes of HD^+^, which agree very well with the chemical intuition that higher temperatures imply more classical behavior, we believe that the obtained results are rather generic. The quantitative values and forms of the regimes will definitely depend on the details of the molecular system under study. Also, the fact that the matter and light subsystems are not in canonical equilibrium is evident. It is, however, useful and physically intuitive to investigate how “noncanonical” these subsystems behave. In contrast to, e.g., considering a RDM of a specific reaction coordinate, there is a simple zero-coupling comparison available, and it is this difference that is the origin of the experimentally observed differences. It is, as pointed out elsewhere,^24,44^ similar to comparing the effect of a solvent to the case of no solvent. Clearly, the coupling to the solvent can change the chemical details of the molecular system, and hence, distinguishing the two cases is important.

However, as was pointed out by several of the authors, the most important situation is the collective coupling regime. With the current computational power, we are not able to solve the ab initio problem of a large molecular ensemble. In contrast to the opinion of some referees, we nevertheless think that a detailed ab initio description of the molecular system under strong coupling conditions is important to understand the changes in the chemical properties. This becomes indeed feasible, at least approximately, if we realize that, already, from the start of the field of polaritonic chemistry, experimentalists have interpreted the collective strong coupling case as also inducing local (single-molecule) strong coupling. In the first review on polaritonic chemistry, Ebbesen writes^20^ “It has been argued that the Rabi splitting experienced by each molecule involved in the collective coupling is not *ℏ*Ω_*R*_ but . If this were the case, the splitting would be tiny, and it is unlikely that any molecular or material property would be modified as observed experimentally.”

This intuition has been theoretically confirmed in different first-principles simulations, e.g., see refs (46), (47) and (50). The basic rationale is that in an intrinsically disordered ensemble, the polarizable ensemble induces strong local fields and thus acts not unlike a highly frequency-dependent solvent.^24,44^ Taking this perspective, we believe that the single-molecule strong coupling results can shed light on the potential changes in the collective coupling regime as well and can also guide us on how to construct more accurate collective coupling models. First of all, the observed loss of light–matter entanglement at low cryogenic temperatures opens the door for efficient numerical partitions into the (only) correlated quantum subsystem. A simple model is suggested by the cavity Born–Oppenheimer picture,^64^ which in its simplest form treats the nuclei and photons classically but can still account for the nonclassical nature of the field fluctuations.^24^ Choosing this semiclassical picture avoids the previously stated issues of mixed quantum statistics. This perspective also agrees with the usual approach to molecular ensembles where the large amount of molecules allows a semiclassical statistical description. For instance, for a large ensemble of gas phase molecules, no permanent dipole appears as the orientations of the dipoles fluctuate randomly.^78^ Also, the coexistence of classical and quantum fluctuations in a Langevin-setting^24^ gives the possibility of stochastic resonances at room temperature by “classical” noncanonical nuclear dynamics. Such stochastic resonances have been proposed as a mechanism to explain the experimentally observed resonances in cavity-mediated chemical reactions,^24^ which emerge in the absence of external periodic driving under ambient conditions^6,10^ but have not yet been fully rationalized theoretically. If we increase the temperature further, we expect that at one point, the classical thermal fluctuations dominate and aforementioned stochastic resonance phenomena are absent. In this case, modifications of ground state chemical processes will most likely be dominated by canonical free energy modifications induced in a cavity due to, e.g., disorder-induced local field effects.^50^ Furthermore, cavity-modified nonadiabatic effects from excited electronic states may also start to play a significant role. Similarly to the high temperature limit, we anticipate canonical nuclear dynamics in the high frequency limit (electronic strong coupling) also, which cannot be accessed with our numerical setup. It is, however, generally assumed that thermal field fluctuations will be negligible under ambient conditions, and thus, only the vacuum mode fluctuations will alter the electronic properties locally^50^ as well as collectively. Therefore, the dynamics of the nuclei will most likely be well described by pure classical canonical dynamics in a thermal equilibrium. This perspective is further supported by recent simulation results, which confirm that nuclear dynamics under electronic strong coupling is well described classically in a Born–Oppenheimer picture with separated electronic and photonic degrees of freedom.^64^

Irrespective of the specific molecular setup, we highlight that the obtained results support the (theoretically^46,47,50^ and experimentally^20,76^) emerging perspective that there are two major classes of effects at play when cavities change chemical or physical properties of matter. On the one hand, there might be genuine changes in the potential energy surface/free-energy landscape. That is, even the temperature-zero ground state is modified. On the other hand, the cavity can modify the exchange of thermal energy with the surrounding environment. Taking into account the fact that the mode structure of free space is intimately connected to the blackbody and thermal radiation, this latter class of effects might be considered trivial. Yet again, site-selective incoherent control over chemical and physical properties would be of practical relevance.

Clearly, our theoretical picture of cavity-modified (non)canonical dynamics of realistic systems is still sketchy at the moment and requires substantial future refinement. In the following, we briefly address two relevant first-principles research directions that we plan to pursue next, based on the findings of the present work:

1. Mixed Quantum Statistics: as described in Section 2.2, we still lack a quantum-statistical equilibrium description of (indistinguishable) matter, which is strongly coupled to photonic modes, for two reasons: first, the strong coupling of the quantized modes to the collective matter dipole a priori hinders the partitioning of the ensemble into weakly interacting entities. Second, if a certain partitioning is assumed, the theoretical treatment of mixed bosonic/Fermionic particle statistics has only been marginally explored so far.^93−95^ Having a thorough quantum-statistical description available will be relevant for the better understanding of molecular polaritonic phases in the ultralow temperature regime, where light–matter entanglement might play a significant role. A detailed understanding of how entanglement is built up in this regime and how mixed quantum statistics might help to protect such entanglement for higher temperatures also is an interesting question to provide robust entangled states. A promising starting point in this direction could be an open quantum systems setting. However, standard open quantum systems methods (e.g., Gorini–Kossakowski–Sudarshan–Lindblad formalism^96−98^) are typically restricted to the dilute gas limit, assuming noninteracting bosonic or distinguishable molecular entities as well as weak coupling to an external bath, i.e., they usually impose Markovian dynamics.^98^ Those assumptions are often not met in practice for realistic molecular systems (e.g., liquids) under strong vibrational coupling conditions, where non-Markovian processes become important. Recently, there have been extensions introduced for non-Markovian dynamics^99,100^ and Fermionic systems,^101,102^ which may help to gain a detailed theoretical understanding of the dynamics of entangled or correlated polaritonic systems with mixed quantum statistics.
2. The Impact of Collective Effects: the presence of multiple (identical) molecules *N* > 1 can significantly enhance the coupling strength of light and matter, which is most prominently identified by a -scaling behavior of the Rabi-splitting in the Dicke model.^103^ While the general relevance of collective scaling effects on various observables in polaritonic systems is undisputed, i.e., they appear in observables that probe the entire system (e.g., optical absorption or nonlinear spectroscopy), little is known about how the collective coupling translates into the individual single-molecule light–matter coupling. It might be that a description based on semiclassical polarizability (e.g., radiation reaction approach) may in principle be sufficient to capture collective effects on chemical reactions at ambient conditions.^104,105^ Such an approach would agree with the usual semiclassical interpretation of molecular ensembles. For a quantum-coherent “supermolecule”, the question of the symmetry of the total ensemble would become important. For instance, quantum-mechanically, small molecules have no permanent dipole since one finds the various nonsymmetric (permanent dipole) solutions superimposed and only the environment favors one over the other. On the other hand, for large molecules or ensembles, the symmetries are only obeyed statistically.^78^ That is, to switch quantum-mechanically between different symmetry states of large molecules becomes more and more unlikely with an increase in the system size. A “supermolecule” could lift such quantum-mechanical switching to macroscopic scales. Our exact results cannot address such collective aspects since we are limited to *N* = 1. However, the loss of light–matter entanglement at low cryogenic temperatures makes the quantum nature of collective strong coupling effects at ambient conditions unlikely. Significant future research effort is needed for a better understanding and description of quantum and classical collective effects in polaritonic molecular ensembles. The possibility of using collectivity at ultralow temperatures to enhance light–matter entanglement is, however, intriguing. Here, the interesting connection to ultracold chemistry seems worthwhile to explore further.^106^

Overall, we think that our ab initio thermal simulation results of a molecular system under vibrational strong coupling conditions have interesting implications for many future theoretical and experimental works in various research disciplines, not only in quantum physics but also in chemistry and materials science in general.

## Supporting Information Available

Numerical methods and additional simulation data (PDF)
