# Supplementary material for: Numerically Exact Solution for a Real Polaritonic System under Vibrational Strong Coupling in Thermodynamic Equilibrium: Loss of Light–Matter Entanglement and Enhanced Fluctuations
Source: J Chem Theory Comput. 2023 Nov 16;19(23):8801–14. doi: 10.1021/acs.jctc.3c00092 (PMC10720342; doi:10.1021/acs.jctc.3c00092)
Supplement: Supplementary file 1 — ct3c00092_si_001.pdf [file ct3c00092_si_001.pdf]

Supporting Information: Numerically exact solution for a real polaritonic system under vibrational strong coupling in thermodynamic equilibrium: Loss of light-matter entanglement and enhanced fluctuations

Dominik Sidler,[*,†,‡] Michael Ruggenthaler,[*,†,‡] and Angel Rubio[*,†,‡,¶,§]

†*Max Planck Institute for the Structure and Dynamics of Matter and Center for Free-Electron Laser Science, Luruper Chaussee 149, 22761 Hamburg, Germany*

‡*The Hamburg Center for Ultrafast Imaging, Luruper Chaussee 149, 22761 Hamburg, Germany*

¶*Center for Computational Quantum Physics, Flatiron Institute, 162 5th Avenue, New York, NY 10010, USA*

§*Nano-Bio Spectroscopy Group, University of the Basque Country (UPV/EHU), 20018 San Sebastián, Spain*

E-mail: dsidler@mpsd.mpg.de; michael.ruggenthaler@mpsd.mpg.de; angel.rubio@mpsd.mpg.de

S1 Numerical Solution of Pauli-Fierz Hamiltonian and Ensemble Averaging

The exact solution of our quantized 3-body matter system coupled to a cavity mode relies on the choice of a highly optimized, i.e. problem specific, coordinate representation,[S1,S2] in combination with Gauss-Laguerre numerical quadrature (see Supporting Information of Ref. S3 for numerical details and S4 of this work for specific computational parameter choices).

When evaluating ensemble averages numerically, we assume $V \to \infty$, which implies a continuum of quantum numbers k_z, which allows an efficient numerical evaluation, i.e. we replace $\sum_{k_z} \mapsto \int_{\infty}^{\infty} dk_z$. Hence, we can apply the Gauss-Hermite quadrature procedure to approximate the infinite integral along k_z numerically, i.e. by using the following relation,[S4]

$$\int_{-\infty}^{\infty} \frac{1}{\sigma\sqrt{2\pi}} e^{-\frac{(y-\mu)^2}{2\sigma^2}} h(y) dy \approx \frac{1}{\sqrt{\pi}} \sum_{i=1}^{s} w_i h(\sqrt{2}\sigma x_i + \mu), \tag{S1}$$

where x_i are the roots of the Hermite polynomial $H_s(x)$ and the weights $w_i = \frac{2^{s-1} s! \sqrt{\pi}}{s^2 [H_{s-1}(x_i)]^2}$.

In our case, we map the ensemble averages of observable $\hat{A}$ as,

$$\begin{aligned}
\langle \hat{A} \rangle_T &= \int_{\infty}^{\infty} dk_z \left(\sum_n \frac{e^{-\beta E_{k_z,n}}}{\mathscr{Z}_{\min}} \left\langle \Phi'_{k_z,n} \middle| \hat{A}' \middle| \Phi'_{k_z,n} \right\rangle \right) \\
&= \int_{\infty}^{\infty} dk_z e^{-\frac{\beta}{2M}\left(k_z - \frac{\lambda_\alpha Q_{\mathrm{tot}} \langle \hat{p}_\alpha \rangle_0}{\omega_\alpha}\right)^2} \left(\sum_n \frac{e^{-\beta E^{\mathrm{red}}_{k_z,n}}}{\mathscr{Z}_{\min}} \left\langle \Phi'_{k_z,n} \middle| \hat{A}' \middle| \Phi'_{k_z,n} \right\rangle \right) \\
&= \int_{\infty}^{\infty} dk_z e^{-\frac{\beta}{2M}\left(k_z - \frac{\lambda_\alpha Q_{\mathrm{tot}} \langle \hat{p}_\alpha \rangle_0}{\omega_\alpha}\right)^2} h(k_z) \\
&\approx \sqrt{\frac{2M}{\beta}} \sum_{i=1}^{s} w_i h\left(\sqrt{\frac{2M}{\beta}} x_i + \frac{\lambda_\alpha Q_{\mathrm{tot}} \langle \hat{p}_\alpha \rangle_{gs}}{\omega_\alpha} \right)
\end{aligned} \tag{S2}$$

and accordingly,

$$\begin{aligned}
\mathscr{Z}_{\min} &= \int_{\infty}^{\infty} dk_z e^{-\frac{\beta}{2M}\left(k_z - \frac{\lambda_\alpha Q_{\text{tot}}\langle\hat{p}_\alpha\rangle_0}{\omega_\alpha}\right)^2} \left(\sum_n e^{-\beta E^{\text{red}}_{k_z,n}}\right) && \text{(S3)}\\
&= \int_{\infty}^{\infty} dk_z e^{-\frac{\beta}{2M}\left(k_z - \frac{\lambda_\alpha Q_{\text{tot}}\langle\hat{p}_\alpha\rangle_0}{\omega_\alpha}\right)^2} g(k_z) && \text{(S4)}\\
&\approx \sqrt{\frac{2M}{\beta}} \sum_{i=1}^{s} w_i g\left(\sqrt{\frac{2M}{\beta}} x_i + \frac{\lambda_\alpha Q_{\text{tot}}\langle\hat{p}_\alpha\rangle_{gs}}{\omega_\alpha}\right). && \text{(S5)}
\end{aligned}$$

This assumes that the transformed $\hat{A}'$ does not depend on k_z, k_y, $\hat{R}_c$ and $\hat{P}_c$, which is indeed the case for all our observables. The COM reduced Energy eigenvalue is defined as $E^{\text{red}}_{k_z,n} := E_{k_z,n} - \big(k_z - \frac{\lambda_\alpha Q_{\text{tot}}\langle\hat{p}_\alpha\rangle}{\omega_\alpha}\big)^2$. Hence, for the determination of $\mu = \frac{\lambda_\alpha Q_{\text{tot}}\langle\hat{p}_\alpha\rangle_0}{\omega_\alpha}$ it is assumed that the ground-state contribution $\langle\hat{p}_\alpha\rangle_0$ to the ensemble average dominates. However, from the symmetry argument in Eq. (19) we immediately notice $\mu = 0$, since $\langle\hat{p}_\alpha\rangle = 0$ for all states in absence of external driving currents, which makes accurate integration possible for few discrete k_z-evaluations only.

S2 Numerics for Logarithmic Negativity

The canonical density matrix given in Eq. (9) can be written for our system in terms of COM, relative and photon basis explicitly as,

$$\hat{\rho} = \sum_{\mathbf{k},n} \frac{e^{-\beta E_{\mathbf{k},n}}}{\mathscr{Z}} \sum_{i,s}\sum_{j,t} c^*_{i,s}(k_z,n) c_{j,t}(k_z,n) \left|e^{-i\mathbf{k}\mathbf{R}_c}\right\rangle |\mathbf{r}_{ci}\rangle_i |q_\alpha\rangle_s \left\langle e^{i\mathbf{k}\mathbf{R}_c}\right| \langle\mathbf{r}_{ci}|_j \langle q_\alpha|_t, \quad \text{(S6)}$$

which accounts for the blockdiagonal nature in terms of COM coordinates. For our subsytem choices $W \in \{\mathbf{r}_{ci}, \hat{q}_\alpha\}$ the partial transpose follows from Eq. (S6) by either $c^*_{i,s}c_{j,t} \mapsto c^*_{j,s}c_{i,t}$ or $c^*_{i,s}c_{j,t} \mapsto c^*_{i,t}c_{j,s}$, leading e.g. to the partial transpose of the photon subsystem,

$$\hat{\rho}'^{\Gamma_{q\alpha}}_\alpha = \sum_{\mathbf{k},n} \frac{e^{-\beta E_{\mathbf{k},n}}}{\mathscr{Z}} \sum_{i,s}\sum_{j,t} c^*_{i,t}(k_z,n) c_{j,s}(k_z,n) \left|e^{-i\mathbf{k}\mathbf{R}_c}\right\rangle |\mathbf{r}_{ci}\rangle_i |q_\alpha\rangle_s \left\langle e^{i\mathbf{k}\mathbf{R}_c}\right| \langle\mathbf{r}_{ci}|_j \langle q_\alpha|_t, \quad \text{(S7)}$$

which can be diagonalised yielding the corresponding eigenvalues $w_{\mathbf{k}',l}^{\Gamma_{q\alpha}}(T) = \frac{e^{-\beta\frac{k_x'^2+k_y'^2+k_z'^2}{2M}}}{\mathscr{Z}} w_{k_z',l}^{\Gamma_{q\alpha}}$ and eigenfunctions $\left|e^{-i\mathbf{k}'\mathbf{R}_c}\right\rangle \sum_{i,s} d_{i,s}^{\Gamma_{q\alpha}}(k_z', l) \left|\mathbf{r}_{ci}\right\rangle_i \left|q_\alpha\right\rangle_s$. For a fixed k_z-value, this diagonalization can efficiently be computed numerically for our HD^+ molecule and the choice of our subsystems. The logarithmic negativity entanglement measure then follows immediatly from:

$$\eta_W(\hat{\rho}) = \log_2\left(\sum_{\mathbf{k},l} |w_{\mathbf{k}',l}^{\Gamma_W}| - w_{\mathbf{k}',l}^{\Gamma_W} + 1\right) \tag{S8}$$

$$= \log_2\left(\int dk^3 \sum_{l} |w_{\mathbf{k}',l}^{\Gamma_W}| - w_{\mathbf{k}',l}^{\Gamma_W} + 1\right), \tag{S9}$$

where in the last step the summation over **k** were approximated by an integral, as it was previously the case for the ensemble averages.

S3 Jaynes-Cummings Light-Matter Entanglement in Canonical Equilibrium

The three energetically lowest eigenfunctions of the JC-model are

$$\left|\Psi_g\right\rangle = |g\rangle \otimes |0\rangle \tag{S10}$$

$$|\Psi_l\rangle = \frac{1}{\sqrt{2}}(|e\rangle \otimes |0\rangle - |g\rangle \otimes |1\rangle) \tag{S11}$$

$$|\Psi_u\rangle = \frac{1}{\sqrt{2}}(|e\rangle \otimes |0\rangle + |g\rangle \otimes |1\rangle) \tag{S12}$$

which are composed from the bare matter $\{|g\rangle, |e\rangle\}$ and photon $\{|0\rangle, |1\rangle\}$ eigenstates, assuming the rotating wave approximation. Furthermore, we have assumed for simplicity that the cavity is tuned exactly on resonance with the first bare matter excitation energy $\omega_\alpha = E_e - E_g$. Expressing the corresponding canonical equilibrium density in the light-matter basis $\{|g\rangle \otimes |0\rangle,\ |g\rangle \otimes |1\rangle,\ |e\rangle \otimes$

$|0\rangle$, $|e\rangle \otimes |1\rangle\}$ leads to the following matrix representation,

$$\rho^{JC} \;=\; \begin{bmatrix} e^{-\frac{E_g}{k_BT}} & 0 & 0 & 0 \\ 0 & \frac{e^{-\frac{E_l}{k_BT}}}{2}+\frac{e^{-\frac{E_u}{k_BT}}}{2} & -\frac{e^{-\frac{E_l}{k_BT}}}{2}+\frac{e^{-\frac{E_u}{k_BT}}}{2} & 0 \\ 0 & -\frac{e^{-\frac{E_l}{k_BT}}}{2}+\frac{e^{-\frac{E_u}{k_BT}}}{2} & \frac{e^{-\frac{E_l}{k_BT}}}{2}+\frac{e^{-\frac{E_u}{k_BT}}}{2} & 0 \\ 0 & 0 & 0 & 0 \end{bmatrix} \tag{S13}$$

with partial transpose

$$\rho^{\Gamma_m,JC} = \begin{bmatrix} e^{-\frac{E_g}{k_BT}} & 0 & 0 & -\frac{e^{-\frac{E_l}{k_BT}}}{2}+\frac{e^{-\frac{E_u}{k_BT}}}{2} \\ 0 & \frac{e^{-\frac{E_l}{k_BT}}}{2}+\frac{e^{-\frac{E_u}{k_BT}}}{2} & 0 & 0 \\ 0 & 0 & \frac{e^{-\frac{E_l}{k_BT}}}{2}+\frac{e^{-\frac{E_u}{k_BT}}}{2} & 0 \\ -\frac{e^{-\frac{E_l}{k_BT}}}{2}+\frac{e^{-\frac{E_u}{k_BT}}}{2} & 0 & 0 & 0 \end{bmatrix}. \tag{S14}$$

The four eigenvalues of the matrix $\rho^{\Gamma_m,JC}$ can be determined and we find three strictly positive eigenvalues and only one negative,

$$\lambda^- = \frac{1}{2}\left(e^{-\frac{E_g}{k_BT}} - \sqrt{e^{-\frac{2E_g}{k_BT}} + \left(e^{-\frac{E_l}{k_BT}} - e^{-\frac{E_u}{k_BT}}\right)^2}\right), \tag{S15}$$

which then solely enters the logarithmic negativity measure.

S4 Simulation Parameters

For the numerical solution, parameters for HD^+ in a cavity were taken from Ref. S3 (e.g. particle mass or radial grid scaling) if not explicitly mentioned otherwise. The number of radial grid points, was slightly reduced from $N_{\mathrm{matter}} = 12$ to $N_{\mathrm{matter}} = 10$, since we required additional resources to explicitly account for the ensemble averaging in k_z (i.e. thermal effects acting on the charged

COM motion). The COM integrals were usually approximated by either $s = 5$ (fluctuations) or $s = 9$ (subsystem temperatures and entanglement) grid points, which effectively required either 3 or 5 evaluations of the computationally expensive $h(k_z)$ expression only. This simplification arises to the ensemble symmetry of k_z with respect to the origin $k_z = 0$, (i.e. $\mu = 0$). The number of Fock states to represent the quantized field was chosen to be $N_{\text{pt}} = 4$ for the fluctuation analysis, $N_{\text{pt}} = 3$ for the subsystem temperature and $N_{\text{pt}} = 2$ for the entanglement measurements. For our chosen coupling strengths a choice $N_{\text{pt}} > 2$ only becomes relevant if one is interested in small deviations of the field fluctuations. Numerical convergence was ensured for all our results.

References

(S1) Hesse, M.; Baye, D. Lagrange-Mesh Calculations of Three-Body Atoms and Molecules. *J. Phys. B: At., Mol. Opt. Phys.* **1999**, *32*, 5605–5617.

(S2) Hesse, M.; Baye, D. Lagrange-Mesh Calculations of Excited States of Three-Body Atoms and Molecules. *J. Phys. B: At., Mol. Opt. Phys.* **2001**, *34*, 1425.

(S3) Sidler, D.; Ruggenthaler, M.; Appel, H.; Rubio, A. Chemistry in Quantum Cavities: Exact Results, the Impact of Thermal Velocities, and Modified Dissociation. *J. Phys. Chem. Lett.* **2020**, *11*, 7525–7530.

(S4) Shizgal, B. A Gaussian quadrature procedure for use in the solution of the Boltzmann equation and related problems. *J. Comput. Phys.* **1981**, *41*, 309–328.

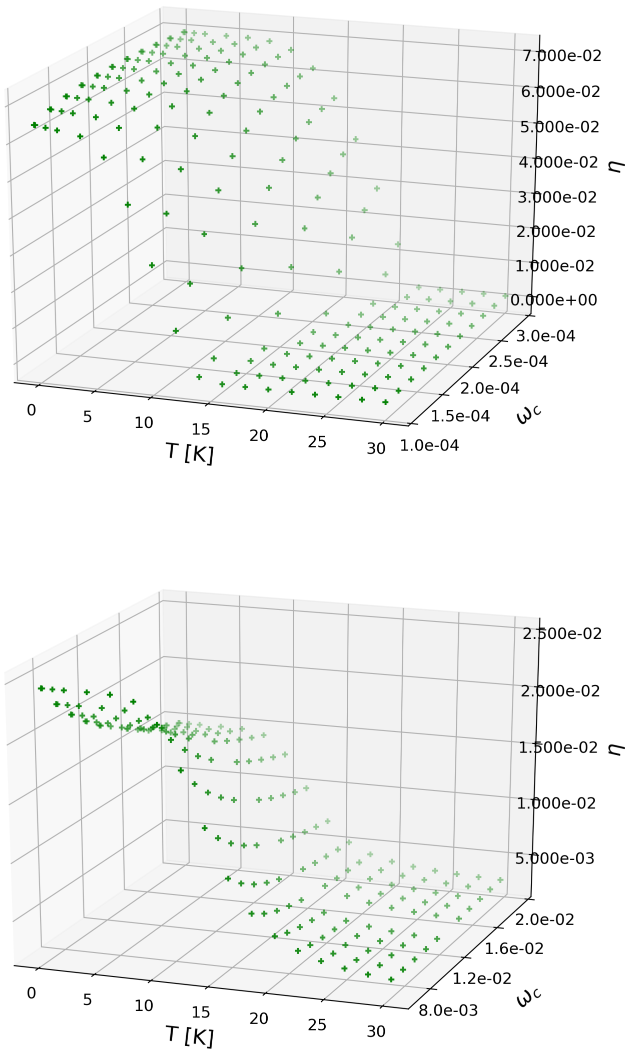

Figure S1: Light-matter entanglement measured by logarithmic negativity η with respect to the temperature T for a coupling constant $\lambda = 0.005$ at different cavity frequencies ω_c (given in atomic units). On the top, the cavity frequency is tuned close to the first ro-vibrational excitation, whereas in the bottom plot the IR to visible regime is covered. In both cases, light-matter entanglement is lost quickly in the deep cryogenic regime. However, while the numerical results for the upper case are certainly converged, deviations may occur in the lower figure due to the highly optimized grid-representation, which was designed to reproduce groundstate and ro-vibrational matter properties with high accuracy, but not simultaneously (!) the higher electronic and vibrational excitations. These states cannot be populated anyways in the chosen temperature regime, but still the accuracy of the matter basis contributions, which are mixed into the hybrid groundstate, may still be reduced in principle. Nevertheless, the loss of light-matter entanglement at low cryogenic temperatures seems to be a generic property for thermal ensembles under strong coupling conditions, roughly independently of the chosen cavity frequency. This is in line with typical experimental evidence, which does not allow for quantum computing devices at sizable temperatures.
